# “Life is Much More Difficult to Manage During Periods”: Autistic Experiences of Menstruation

**DOI:** 10.1007/s10803-018-3664-0

**Published:** 2018-07-07

**Authors:** Robyn Steward, Laura Crane, Eilish Mairi Roy, Anna Remington, Elizabeth Pellicano

**Affiliations:** 1 Centre for Research in Autism and Education (CRAE), UCL Institute of Education, University College London, London, UK; 2 Department of Educational Studies, Macquarie University, 29 Wally’s Walk, Sydney, 2109 Australia

**Keywords:** Autism, Women’s health, Menstruation, Menarche, Self-regulation, Sensory sensitivities

## Abstract

Although menarche and menstruation are perceived to be overwhelmingly negative events for developmentally-disabled women, women’s health issues remain under-researched in autism. Here, we conducted a preliminary investigation of the experiences of post-menarcheal autistic (n = 123) and non-autistic (n = 114) respondents to a brief online survey. Although autistic respondents reported many overlapping issues and experiences with non-autistic respondents, they also highlighted distinct—and sometimes-distressing—issues relating to menstruation, especially a cyclical amplification of autistic-related challenges, including sensory differences and difficulties with regulating emotion and behavior, which had a significant, negative impact on their lives. These initial findings call for systematic research on the potential causes, correlates and consequences of menstrual-related problems in autistic individuals—across the spectrum and the lifespan.

## Introduction

The onset of menses (menarche) is an important—and often challenging (Burrows and Johnson 2005)—transition in any woman’s life. This is especially the case for developmentally-disabled women, who may experience menarche and menstruation differently—and more negatively—compared to non-disabled women (Ditchfield and Burns 2004; Rodgers and Lipscombe 2005). These include frequent reports of dysmenorrhoea (painful periods), menorrhagia (heavy periods), menstrual hygiene issues and cyclical mood and behavioral changes, akin to premenstrual syndrome (PMS) [and its more severe form, premenstrual dysphoric disorder; American Psychiatric Association (APA) 2013] (Burke et al. 2010; Jeffery et al. 2013; Mason and Cunningham 2008; Rodgers et al. 2006).

Remarkably little is known, however, about the menstrual experiences of women on the autism spectrum. This paucity of research may be unsurprising given the male predominance in autism (see Loomes et al. 2017) but the few existing studies in this area give cause for concern. Although there are apparently no significant differences in the age of menarche between autistic girls and girls with other developmental conditions (Burke et al. 2010), there are several reports (including case studies) of marked changes linked to menarche and menstruation in autistic girls and women (the majority with additional intellectual disabilities), including cyclical self-injurious behaviors (Lee 2004), mood symptoms and emotional dysregulation (Burke et al. 2010; Hamilton et al. 2011; Lee 2004; Obaydi and Puri 2008), and an amplification of autistic symptoms (sensory issues and repetitive behaviors; Hamilton et al. 2011; Lee 2004).

One observational study with women with additional intellectual disabilities living in residential homes and care units in England reported an alarming result: of the 26 autistic women sampled, 92% (n = 24) fulfilled DSM-IV (APA 2000) criteria for late luteal phase dysphoric disorder (a severe form of PMS), compared with only four (11%) of the 36 non-autistic women (Obaydi and Puri 2008). The lack of research on, and awareness of, these purportedly higher rates of premenstrual symptoms in autistic women means both that their potential cause(s) remain unknown and that, worryingly, autistic women are unlikely to receive the gynaecological care they may need. Moreover, to our knowledge, no existing study has directly examined whether these apparent premenstrual symptoms are a problem for the person concerned, *from their own perspective*.

This preliminary study—co-produced by an autistic woman (RS) and non-autistic female researchers (LC, ER, AR, LP)—sought to redress the imbalance in research, by asking autistic and non-autistic people about their experiences of menstruation through an online survey. Specifically, we sought to understand the kind of information they would have liked to have known at menarche, and whether, for autistic participants specifically, they felt being autistic affected, or was affected by, menstruation and its manifestations.

## Method

The survey began with a series of background items, including participants’ age, gender and connection with autism. These items were followed by questions on their experiences of menstruation and of growing up more broadly. The three most relevant to the current report are analysed below,^1^ including (1) “How did you first learn about periods?” (closed question), (2) “What information do you think would have been important to have before you started your period?” (open question), and (3) “Do you think that you have experienced difficulties with periods that are related to autism?” (open question).

Participants were recruited via a convenience sampling method (using website posts and social media). In all, 459 people completed the survey. People were allowed to identify more than one connection—as being autistic themselves, a parent, a professional or a sibling—to reflect potential multiple roles, but were also asked to identify the perspective from which they responded to the survey (e.g., as autistic). For this report, we focus on those participants who identified themselves to be ‘formally diagnosed as autistic’ (n = 144)^2^ or ‘non-autistic’ (n = 132). Of these 276 participants, 39 reported never having experienced periods (n = 11) or did not answer all relevant questions (n = 28) and were excluded from the dataset. Subsequent analyses were therefore based on complete responses from 237 participants (autistic: n = 123; non-autistic: n = 114), ranging in age from 16 to over 60 years (see Table 1 for participant details).

**Table 1:** Background information for respondents to the online survey for each (autistic, non-autistic) group

	Autistic (n = 123)	Non-autistic (n = 114)
Age range (in years)		
16–18	9	6
19–25	33	26
26–31	33	20
32–45	36	44
46–59	12	13
60+	0	5
Gender^a^		
Female (including transgender women)	83	96
Male (including transgender men)	7	0
Non-binary	26	15
Other	6	3
Prefer not to say	1	0
Also identified as a:		
Parent	14	18
Professional	14	16
Sibling	13	5

^a^Gender categories were identified in direct consultation with the autistic community

The survey took approximately 10 min to complete and was hosted by SurveyMonkey between February and August 2016. All participants gave informed consent to take part prior to participation. Ethical approval for this study was granted by the Research Ethics Committee at UCL Institute of Education, University College London (REC 874).

### Data Analysis

Descriptive results for the initial, closed questions are presented first. Next, we analysed the qualitative responses across the two open-ended questions using thematic analysis (Braun and Clarke 2006). We used an inductive (“bottom up”) approach, providing descriptive overviews of the key features of the semantic content of the data within an essentialist framework. We independently familiarised ourselves with the data, and met several times to agree the initial codes, review the results, resolve discrepancies and decide on final themes and subthemes.

## Results

### Quantitative Results

The majority of participants first obtained information about periods through either their parents or through school (see Table 2), with some finding out about menstruation from friends and printed material, and, for a handful of people, from medical professionals or the internet. This pattern was similar across groups, with the exception of fewer autistic women discovering information from friends than non-autistic women.

**Table 2:** Participants’ responses to the question, “How did you first learn about periods?”

	Autistic (n = 123)	Non-autistic (n = 114)
The internet	3	3
Friends	13	26
Parents	70	66
School	55	46
Doctor or other medical professional	1	5
I don’t know	6	6
Other	19	25

The total numbers exceed the number of participants in each group because participants could endorse more than one category. Most of the responses in the ‘other’ category related to printed material (books, magazines or leaflets), “my sister” or “when it happened”

### Qualitative Results

**Fig. 1:**
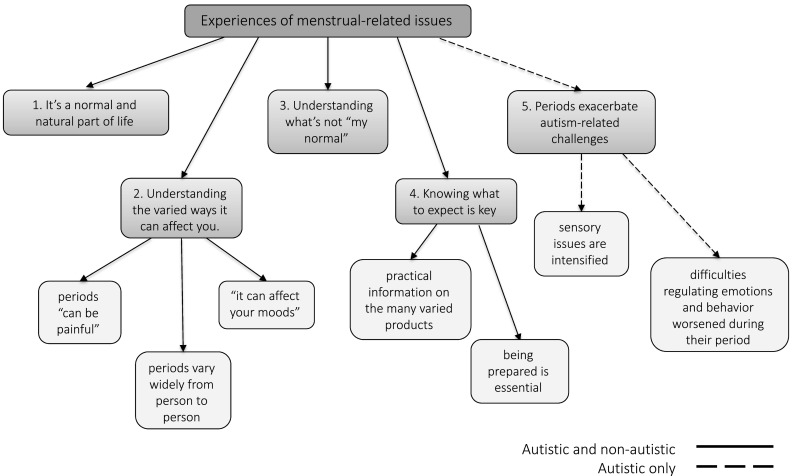
Respondents’ experiences of menstrual-related issues: themes and subthemes

Figure 1 displays themes and subthemes identified from the analysis. For the sake of brevity, we report the themes below collapsed across both groups (subthemes are italicised). When attributing quotes, ‘A’ refers to autistic respondents, ‘NA’ to non-autistic respondents. Participant numbers are included to illustrate the breadth of responses.

#### It’s a Normal and Natural Part of Life

Respondents spoke of the taboo associated with having periods. They emphasised the need to reassure young people that periods are “not dirty” (A23) or “something to fear or be ashamed of” (NA36) or “embarrassed by” (NA65); rather, they are a “healthy” (NA96), “natural” (A6) and “normal part of growing up” (NA68). They also felt that it was important for everyone—even those who have not experienced periods—to understand this, so that they “understand what their mum/sister/etc. is going through” (A71). Both autistic and non-autistic respondents further reported that young people need to understand “how their body works and why” (NA94), including biological information about “how the menstrual cycle and organs work, and the anatomy of the vagina/vulva/uterus etc.” (NA59), and “why [periods] happen” (A57). Autistic respondents in particular wanted “more detail about this” (A73).

#### Understanding the Varied Ways it Can Affect You

Respondents highlighted the need for young people to know “what happens before their first period” (A8) so that it “doesn’t come as a terrifying surprise” (NA16). In particular, they should understand that the cramps that come with *periods “can be painful”* (NA48), and that there can be other physical symptoms, too, including bloating (water retention), “tender breasts” (A22), “skin changes” (A110) or acne, changes to eating habits, constipation, and “the way it affects your weight” (NA60). Several autistic respondents noted specifically that “it was helpful to know beforehand that I wasn’t dying” (A7) from the bleeding and/or pain.

Respondents also cited the many ways that *“it can affect your moods”* (NA60)—“before and possibly after your period, not just during” (A29)—and that these changes “are normal” (A66). Some respondents noted the importance of providing an explanation of these “mood swings” (A78) and “the reason one is acting in a particular way” (NA52). Indeed, one autistic participant described, “about once a month, I get anxious and melancholic for no reason. This mood lasts for a day or two, at which point my period arrives, and my ‘normal’ mood resumes. Understanding that about myself makes that melancholy a lot more bearable/manageable and helps my partner understand my behavior/mood” (A52).

Both autistic and non-autistic participants emphasised the need to improve knowledge on “how long it lasts, how often it happens, how heavy/light it will be” (A84) but, critically, that *periods vary widely from person to person*. Indeed, they felt that knowing that “everyone gets them differently” (A85) would have been helpful for them to understand “that what’s normal for me is not what’s normal for everyone else” (NA41). One autistic respondent summed it up: “Some people get them heavy, some people get them light, not everybody gets them regularly, not everybody gets them exactly 28 days apart, some people get pains, some people get moody, sometimes medical conditions make them irregular, some people get them for more than a week at a time, some people get them for only a couple of days” (A85).

#### Understanding What’s Not “My Normal”

Respondents felt that it was important to “pay attention to what normal is for me, and to know how to react if my normal changes” (NA41). They spoke of the need for young people to “know how to track their cycles effectively so that they can be aware of how their body is … and be prepared for any irregularities that might occur” (A22), including the amount of pain, the amount of blood and the frequency between periods. They stressed the need to know how to distinguish between, for example, “normal period cramps and unusually painful periods” (NA60) to identify “how much is acceptable before you need to worry” (NA71) and “what could be a warning sign of a medical problem” (A65).

#### Knowing What to Expect is Key

Respondents suggested that young people needed *practical information on the many varied products* to use, including a “chance to familiarize themselves with a variety of products before they start” (A50), information on “how to choose menstrual hygiene products” (NA19), where to get them, and “disposal methods” (A28). They stressed that introductory information should be accessible, “breaking down initial concepts” (A15) with a “step-by-step ‘this is how you deal’ instructions and tips” (A42), particularly for young autistic people. They also wanted young people to know about the different strategies available to relieve pain (including medication), and how to deal with mood swings and other symptoms or issues like “acne, cramps, bloating, nausea” (NA35).

Respondents also noted that *being prepared is essential*. This preparation could include a “script for what to say to a nurse or parent when you start your first one or are unprepared” (A50), knowing “what to do at school, if you are out in a public place, if you are on holiday” (NA113) and having an “action plan for mishaps (stains, stains in public places, forgot supplies, etc.)” (NA50). They also wanted to reassure them that “it’s ok to tell your teachers if you need more time for the bathroom” (NA38) and that it was important to identify “who to ask questions about periods” (NA13) and who to turn to for support, especially “if you might feel there is a problem” (A104).

#### Periods Exacerbate Autism-Related Challenges

Although some participants were unsure whether their menstrual experiences were related to being autistic (“I have only ever been an autistic person having a period!”; A80), many autistic participants felt that their “symptoms worsen dramatically” (A47), often making “life much more difficult to manage during periods” (A93). One participant stated: “autism does play a role. It can become much more overwhelming and harder to maintain control of the things that already take a lot of effort for us to keep on top of, during a period” (A13). Participants highlighted sensory and self-regulation difficulties in particular.

Respondents felt that *sensory issues are intensified* during menstruation, as described above. For the most part, these related to pre-existing hypersensitivities becoming “extra sensitive during my period” (A107), such that “everything is magnified when it’s that time of the month” (A78). Participants described “being sensitive to the smell of the blood” (A17), “finding my skin and body more sensitive in general” (A43), being “more sensitive and reactive to noise, touch and visual stimuli” (A99), and “struggling the most with the physical pain from cramps” (A22). Pain could be particularly difficult to bear: “When it’s at its worst, I find myself unable to focus well because all I can focus on is the ache and the sense of where in my body that pain lives” (A44). Participants also reported how these hypersensitivities and the “sensory overload [that] happens far more frequently just before and during a period” (A103) further exacerbated other autistic experiences, including “dealing with unrelated problems caused by my autism (harder to filter noise etc.)” (A28) and being “more prone to self-injurious behavior” (A94).

Participants also highlighted how *difficulties regulating emotions and behavior worsened during their period*. Some noted that “executive dysfunction gets worse when I have cramps” (A98), which “made dealing with periods difficult—keeping clean and changing pads” (A74). They also highlighted difficulties “recognising and managing my emotions, which is amplified just before and during my periods” (A45) and even “an inability to describe my emotions while experiencing PMS” (A41). One participant explained: “understanding my own emotions has always been difficult for me so any mood swings made life even more difficult” (A17). They also reported that exaggerated difficulties often led to “heightened anxiety” (A99) and, most commonly, meltdowns: “I have more meltdowns, and worse meltdowns, just before my period” (A30). One participant also noted that their epilepsy was affected, with increased seizures during menstruation. These cyclical symptoms were often so severe that participants sought (usually hormonal) medication to manage them.

## Discussion

This preliminary study directly elicited, for the first time, autistic people’s views and experiences on menarche and menstruation. Notably, autistic and non-autistic respondents cited many similar issues, including needing to understand what was “normal” in terms of menstrual cycle length, amount and duration of flow, the often-dramatic effects that menstruation can have on mood, behavior and bodily symptoms and, importantly, what was normal for them. The importance of educating young people and their parents in what to expect at menarche has been emphasized previously (American Academy of Pediatrics 2006) but rarely so for young autistic people, who may be at increased risk for serious premenstrual-related disorders (Obaydi and Puri 2008). The current study suggests that, consistent with the albeit-limited research (Burke et al. 2010; Hamilton et al. 2011; Lee 2004; Obaydi and Puri 2008), autistic people’s menstrual experiences are in some ways distinct from those of non-autistic people, placing extra strain on what can be already-challenging lives.

Indeed, for the autistic participants sampled here, menstruation was seen as a particularly difficult and distressing event (akin to women with other developmental disabilities; Ditchfield and Burns 2004), during which their pre-existing challenges—especially regarding sensory hypersensitivies and difficulties with regulating emotions and behavior—become exacerbated before, during and after menses. The amplification of pre-existing autistic features has been reported previously (e.g., Hamilton et al. 2011; Lee 2004) but no study has sought to understand the impact of menstruation on the individuals themselves. Those sampled here described overwhelmingly negative experiences, especially exaggerated sensory issues and intensified executive and emotion-regulation problems, which had often-serious consequences, including “shutdown”, withdrawal and heightened anxiety—and therefore reduced participation in work, social and community life. Understanding the prevalence of premenstrual-related symptoms in individuals across the autism spectrum, the causes of such symptoms (which may be related to higher levels of hormonal fluctuations; Obaydi and Puri 2008), and their associated impact (including the potential treatment side-effects of cyclical changes) is critical for further research.

One important step to mitigate potential problems following menarche is to increase knowledge of menstrual-related issues in young people and their parents (see American Academy of Pediatrics 2006), particularly in the form of accessible, step-by-step guides and strategies for how to deal with pain and mood changes in particular (see Steward, in press). This is especially important for young autistic people, who may be less likely to gather information about sexual topics from informal social settings (with peers—as evidenced in Table 2), whose parents might be reticent to discuss puberty and sexual health and may begin these conversations later (Pownall et al. 2012; Sedgewick et al. 2018; Cridland et al. 2014, for discussion), and whose clinicians may fail to notice (or prioritize) any link between menstruation and mood- or behavior-related features (Kaminer et al. 1988). The current absence of this knowledge rather worryingly means that the particularly severe symptoms reported by some girls and women may be going unrecognised by clinicians and therefore not treated appropriately.

Given the nature of the current methodology, it was possible neither to confirm, for self-declared autistic respondents, where they lie on the autism spectrum nor to ensure that we did not oversample those with particularly problematic menstrual experiences. Notwithstanding, these preliminary findings serve to stress the importance of these issues for autistic people and call for greater attention on women’s health issues across the lifespan, including systematic investigations on the causes, correlates and consequences of menstruation (particularly with regard to mental health) for autistic young people and adults—from their own perspectives and the perspectives of supportive others (parents, teachers).

## Footnotes

1 Two further questions were asked, relating to growing up more generally and about strategies for helping school-age children to access the toilet during their period. These questions were not analyzed in this current study, but were designed for use by the first author, to inform the content of a forthcoming book (Steward, in press).

2 Note that some individuals reported that they either were going through the process of a clinical diagnosis (n = 32) or were self-diagnosed as autistic (n = 88). These participants were excluded from all analyses.
